# Synthesis and *in vivo* anti-ulcer evaluation of some novel piperidine linked dihydropyrimidinone derivatives

**DOI:** 10.1080/14756366.2018.1474212

**Published:** 2018-05-24

**Authors:** Mashooq Ahmad Bhat, Mohamed A. Al-Omar, Ahmed M. Naglah

**Affiliations:** a Department of Pharmaceutical Chemistry, College of Pharmacy, King Saud University, Riyadh, Saudi Arabia;; b Department of Pharmaceutical Chemistry, Drug Exploration and Development Chair (DEDC), College of Pharmacy, King Saud University, Riyadh, Saudi Arabia;; c Peptide Chemistry Department, Chemical Industries Research Division, National Research Centre, Cairo, Egypt

**Keywords:** Dihydropyrimidinone, piperidine, anti-ulcer, cytoprotective

## Abstract

Dihydropyrimidinone derivatives containing piperidine moiety were synthesised in a good yield. All the compounds were confirmed by elemental analysis and spectral data. Anti-ulcer activity of novel dihydropyrimidinone-piperidine hybrids (**1–18**) was evaluated. Among them, four compounds (**3**, **8**, **11** and **15**) were found to be most active in 80% ethanol-induced ulcer experimental animal model. All the potent compounds were further evaluated for anti-ulcer activity by different *in vivo* anti-ulcer models to study the effect of compounds on anti-secretory and cytoprotective activities. All the active compounds inhibited the formation of gastric ulcers and increased the formation of gastric mucin secretion. Compound **15** was found to be the most potent compound of the series as anti-ulcer agent. Additional experimental studies on lead compound **15** will result in a new class of orally active molecule for anti-ulcer activity.

## Introduction

Peptic ulcer disease (PUD) is prevalent in large population of the world. The gastric mucosal ulcer, occurring from an imbalance between the gastro protective factors (e.g. prostaglandin, mucin, bicarbonate, blood supply and nitric oxide) and the aggressive factors (e.g. pepsin and gastric acid), presents in the gastric mucosa^1^^,^^2^. The risk factors of getting PUD include *Helicobacter pylori* infection, frequent use of pain killer medication and stress-induced gastric mucosal lesions^3^. The anti-ulcer drugs act by decreasing the secretion of gastric acid and/or increasing the defence system by increasing the mucin secretion. The anti-secretory drugs include ranitidine, a histamine H_2_ receptor antagonist; omeprazole, irreversible proton pump inhibitor and antacids. These drugs treat PUD by reducing or neutralising the gastric acid^4^. Drug tolerance has been reported during drug therapy of PUD by conventional drugs. Also, these drugs have serious side effects when used for a long time, which include hypergastrinemia, osteoporosis, development of carcinoids and increased risk of bacterial infection. Sucralfate is used for the treatment of gastric ulceration, but does not show good results for the ulceration caused by non-steroid anti-inflammatory drugs (NSAIDs)^5^. NSAIDs associated ulcers can be prevented by misoprostol (analogue of prostaglandin E_1_), but is limited by abnormal side effects^6^. Therefore, there is a need for novel and potent anti-ulcer agents with improved safety profile.

Pyrimidines have played an important role in the field of medicinal chemistry^7^. Pyrimidines are important scaffold in medicinal chemistry, because of their potential biological activities such as anti-tumour, anti-viral and anti-bacterial^8–10^. Some of them have been used as potential anti-hypertensive agents. 4-Aryl-1,4-dihydropyridines like nifedipine was first introduced as antihypertensive in 1975. Dihydropyridines are the most effective calcium channel blockers used for various cardiovascular diseases^11^. Anti-ulcer activities have been reported for several calcium channel blockers including nifedipine^12^. It is thus assumed that structural analogues of nifedipine may possess anti-ulcer potential. Dihydropyrimidines, popularly known as Biginelli’s compounds, are associated with broad spectrum of biological activities^13^^,^^14^. Derivatives of dihydropyrimidine have been reported to possess potent anti-ulcer and anti-secretory activity^15^^,^^16^.

Piperidine is an organic compound with the molecular formula (CH_2_)_5_NH. This heterocyclic amine consists of a six-membered ring. Piperidine is an important pharmacophore in the field of medicinal chemistry. It is reported to have various pharmacological activities^17–20^. Piperidine derivatives are also reported to have anti-secretory and anti-ulcer activity^21^^,^^22^.

The literature study revealed that compounds containing these two important moieties (dihydropyrimidinone and piperidine) may have potential for the treatment of PUD. Hybrid approach, in the drug design, involves the addition two different pharmacophoric moieties to produce hybrid molecules with improved efficacy. In the present study, a series of novel dihydropyrimidinone and piperidine scaffold hybrids were synthesised, characterised by spectral data and screened for their gastric anti-ulcer activity in several *in vivo* ulcer models.

## Experimental

### Chemistry

#### Materials and methods

Ultraviolet light was used for the visualisation of thin layer chromatography (TLC) spots. Spectrum BX, PerkinElmer FT-IR spectrophotometer was used for performing FTIR. Gallenkamp melting point apparatus was used for performing melting points, which was uncorrected. Bruker NMR 500 MHz and 125 MHz spectrophotometer were used for ^1^H and ^13^C NMR. All the samples were processed in DMSO-d_6_ with tetramethylsilane as an internal standard. Molecular masses of all the compounds were measured by mass spectroscopy. CHN Elementar (Analysensysteme GmbH, Germany) was used for the elemental analysis of the compounds. The X-ray diffraction measurements were made using Bruker (2009) (Bruker AXS Inc., Madison, WI), at wavelength λ = 10,554,184 Å. Crystallographic data for compounds **(III)** and **13** have been deposited with Cambridge Crystallographic Data Center (CCDC) under numbers 1532826 and 1532825, respectively. Copies of the data can be obtained, free of charge, on application to CCDC, 12 Union Road, Cambridge CB2 1EZ, UK [Fax: +44-1223-336033; email: deposit@ccdc.cam.ac.uk or http://www.ccdc.cam.ac.uk].

##### Synthesis of 3-(dimethylamino)-1-[4-(piperidin-1-yl) phenyl]prop-2-en-1-one (III)

A mixture of 1-[4-(piperidin-1-yl) phenyl]ethan-1-one **(I)** (0.02 mol) and dimethylformamide-dimethylacetal (DMF-DMA) **(II)** (0.023 mol) was refluxed for 10 h without solvent on a heating mantle, the reaction mixture was left to cool slowly. The precipitate was obtained. Diethyl ether was added to the precipitate and filtration was performed under vacuum. The obtained product was recrystallised from absolute ethanol. Yield: 90%; m.p.: 150–152 °C; IR (KBr) cm^−1^: 2800 (ArC-H), 1675 (C = O), 1636 (C = O), 1618 (C = C); ^1^H NMR (500 MHz, DMSO-d_6_): *δ* = 1.5 (6H, s, 3 × –CH_2_, *piperidine*), 2.89 (3H, s, NCH_3_), 3.09 (4H, s, 2 × –CH_2_, *piperidine*), 3.17 (3H, s, NCH_3_), 5.79 (1H, d, *J* = 12.5 Hz, = CH), 6.91 (2H, t, *J* = 9.0 Hz, Ar-H), 7.65 (1H, d, *J* = 12.5 Hz, =CH), 7.78 (2H, d, *J* = 8.5 Hz, Ar-H); ^13^C NMR (125.76 MHz, DMSO-d_6_): *δ* = 24.4, 25.4, 48.8, 91.1, 113.9, 129.3, 129.6, 163.4, 163.5, 188.0; MS: *m/z* = 258.30 [M]^+^; analysis for C_16_H_22_N_2_O: C (74.38) H (5.58) N (10.84)%; found C (74.10) H (5.56) N (10.81)%.

##### General synthesis of 4-(substituted phenyl)-5-[4-(piperidin-1-yl) benzoyl]-3,4-dihydropyrimidin-2(1H)-one (1–18)

A mixture of enaminone (**III**) (0.01 mol), differently substituted benzaldehyde (0.01 mol), urea (0.01 mol) and glacial acetic acid (10 ml), was refluxed for 3 h. The precipitates (**1–18)** were obtained by pouring the reaction mixture into the ice–cold water. The products were obtained by filtration under vacuum. The products were washed several times with water. The obtained products were recrystallised from glacial acetic acid.

*4-Phenyl-5-[4-(piperidin-1-yl) benzoyl]-3, 4-dihydropyrimidin-2(1H)-one***(1)**: colour: yellow; yield: 50%; m.p.: 220–222 °C; UV *λ*max (methanol) = 404 nm; IR (KBr) cm^−1^: 3273 (N–H), 2800 (ArC-H), 1675 (C = O), 1636 (C = O), 1618 (C = C); ^1^H NMR (500 MHz, DMSO-d_6_): *δ* = 1.56 (8H, s, 4 × –CH_2_, *piperidine*), 2.74 (1H, s, –CH, *piperidine*), 2.89 (1H, s, –CH, *piperidine*), 5.46 (1H, s, H-4), 6.9 (2H, d, *J* = 8.5 Hz, Ar-H), 7.0 (1H, s, NH, D_2_O exchange), 7.25–7.43 (7H, m, Ar-H), 7.78 (1H, s, =CH), 9.18 (1H, s, –CONH, D_2_O exchange); ^13^C NMR (125.76 MHz, DMSO-d_6_): *δ* = 24.4, 25.3, 31.2, 36.2, 48.0, 48.5, 48.6, 54.1, 113.0, 113.8, 126.8, 127.2, 127.7, 128.9, 130.7, 139.3, 144.7, 152.0, 153.6, 162.7, 190.5; MS: *m/z* = 360.79 [M]^+^; analysis for C_22_H_23_N_3_O_2_: C (73.11) H (6.41) N (11.63)%; found C (73.39) H (6.43) N (11.60)%.

*4-(2-Nitrophenyl)-5-[4-(piperidin-1-yl) benzoyl]-3,4-dihydropyrimidin-2(1H)-one***(2)**: colour: brown; yield: 60%; m.p.: 190–192 °C; UV *λ*max (methanol) = 426 nm; IR (KBr) cm^−1^: 3443 (N–H), 2852 (ArC-H), 1634 (C = O), 1595 (C = O), 1567 (C = C); ^1^H NMR (500 MHz, DMSO-d_6_): *δ* = 1.54 (8H, s, 4 × –CH_2_, *piperidine*), 3.44 (1H, s, –CH, *piperidine*), 3.48 (1H, s, –CH, *piperidine*), 6.11 (1H, s, H-4), 6.89 (2H, d, *J* = 9.0 Hz, Ar-H), 7.14 (1H, s, NH, D_2_O exchg.), 7.38–7.89 (8H, m, Ar-H), 8.10 (1H, s, =CH), 9.42 (1H, s, –CONH, D2O exchange); ^13^C NMR (125.76 MHz, DMSO-d_6_): *δ* = 19.0, 24.4, 25.3, 48.5, 50.1, 56.5, 1117.7, 123.8, 124.4, 126.7, 129.1, 130.0, 130.7, 134.3, 138.8, 140.3, 148.3, 151.2, 153.6, 190.1; MS: *m/z* = 403.80 [M-2]^+^; analysis for C_22_H_22_N_4_O_4_: C (65.01) H (5.46) N (13.78)%; found C (65.26) H (5.47) N (13.73)%.

*4-(4-Nitrophenyl)-5-[4-(piperidin-1-yl) benzoyl]-3,4-dihydropyrimidin-2(1H)-one***(3)**: colour: yellow; yield: m.p.: 180–182 °C; UV *λ*max (methanol) = 405 nm; IR (KBr) cm^−1^: 3273 (N–H), 2800 (ArC-H), 1675 (C = O), 1636 (C = O), 1618 (C = C); ^1^H NMR (500 MHz, DMSO-d_6_): *δ* = 1.55 (8H, s, 4 × –CH_2_, *piperidine*), 2.73 (1H, s, –CH, *piperidine*), 2.89 (1H, s, –CH, *piperidine*), 5.58 (1H, s, H-4), 6.89 (2H, d, *J* = 9.0 Hz, Ar-H), 7.09 (1H, s, NH, D_2_O exchange), 7.41–7.93 (8H, m, Ar-H), 8.21 (1H, s, =CH), 9.35 (1H, s, –CONH, D_2_O exchange); ^13^C NMR (125.76 MHz, DMSO-d_6_): *δ* = 19.0, 24.4, 25.3, 48.5, 54.0, 56.5, 111.9, 113.8, 124.2, 126.9, 128.2, 130.7, 140.1, 147.1, 151.7, 151.8, 153.6, 190.2; MS: *m/z* = 406.00 [M]^+^; analysis for C_22_H_22_N_4_O_4_: C (65.01) H (5.46) N (13.78)%; found C (65.25) H (5.46) N (13.72)%.

*4-(3-Nitrophenyl)-5-[4-(piperidin-1-yl) benzoyl]-3,4-dihydropyrimidin-2(1H)-one***(4)**: colour: yellow; yield: m.p.: 185–187 °C; UV λmax (methanol) = 404 nm; IR (KBr) cm^−1^: 3256 (N–H), 2800 (ArC-H), 1701 (C = O), 1685 (C = O), 1654 (C = C); ^1^H NMR (500 MHz, DMSO-d_6_): *δ* = 1.55 (8H, s, 4 × –CH_2_, *piperidine*), 2.73 (1H, s, –CH, *piperidine*), 2.89 (1H, s, –CH, *piperidine*), 5.58 (1H, s, H-4), 6.89 (2H, d, *J* = 9.0 Hz, Ar-H), 7.09 (1H, s, NH, D_2_O exchange), 7.41–7.93 (8H, m, Ar-H), 8.21 (1H, s, =CH), 9.35 (1H, s, –CONH, D_2_O exchange); ^13^C NMR (125.76 MHz, DMSO-d_6_): *δ* = 19.0, 24.4, 25.3, 48.5, 54.0, 56.5, 111.9, 113.8, 124.2, 126.9, 128.2, 130.7, 140.1, 147.1, 151.7, 151.8, 153.6, 190.2; MS: *m/z* = 406.21 [M]^+^; analysis for C_22_H_22_N_4_O_4_: C (65.01) H (5.46) N (13.78)%; found C (65.24) H (5.45) N (13.71)%.

*4-(4-Chlorophenyl)-5-[4-(piperidin-1-yl) benzoyl]-3,4-dihydropyrimidin-2(1H)-one***(5)**: colour: yellow; yield: 70%; m.p.: 230–232 °C; UV *λ*max (methanol) = 421 nm; IR (KBr) cm^−1^: 3261 (N–H), 2931 (ArC-H), 1654 (C = O), 1636 (C = O), 1600 (C = C); ^1^H NMR (500 MHz, DMSO-d_6_): *δ* = 1.57 (8H, s, 4 × –CH_2_, *piperidine*), 2.73 (1H, s, –CH, *piperidine*), 2.89 (1H, s, –CH, *piperidine*), 5.44 (1H, s, H-4), 6.9 (2H, d, *J* = 7.0 Hz, Ar-H), 7.0 (1H, s, NH, D_2_O exchange), 7.33–7.40 (6H, m, Ar-H), 7.81 (1H, s, =CH), 9.34 (1H, s, –CONH, D_2_O exchange); ^13^C NMR (125.76 MHz, DMSO-d_6_): *δ* = 24.4, 25.3, 31.2, 36.2, 48.5, 53.6, 112.5, 113.8, 127.1, 128.7, 128.8, 130.7, 132.3, 139.6, 143.7, 151.9, 153.6, 190.4.; MS: *m/z* = 395.82 [M]^+^; analysis for C_22_H_22_ClN_3_O_2_: C (66.75) H (5.60) N (10.61)%; found C (66.50) H (5.61) N (10.62)%.

*4-(2,4-Dichlorophenyl)-5-[4-(piperidin-1-yl) benzoyl]-3,4-dihydropyrimidin-2(1H)-one***(6)**: colour: yellow; yield: 75%; m.p.: 195–197 °C; UV *λ*max (methanol) = 406 nm; IR (KBr) cm^−1^: 3273 (N–H), 2800 (ArC-H), 1671 (C = O), 1630 (C = O), 1615 (C = C); ^1^H NMR (500 MHz, DMSO-d_6_): *δ* = 1.55 (8H, s, 4 × –CH_2_, *piperidine*), 3.2 (2H, s, –CH, *piperidine*), 5.83 (1H, s, H-4), 6.89 (2H, d, *J* = 8.5 Hz, Ar-H), 7.10 (1H, s, NH, D_2_O exchange), 7.39–7.56 (7H, m, Ar-H), 7.75 (1H, s, =CH), 9.32 (1H, s, –CONH, D_2_O exchange); ^13^C NMR (125.76 MHz, DMSO-d_6_): *δ* = 19.0, 24.4, 25.3, 48.5, 52.4, 56.5, 111.0, 113.8, 127.6, 128.1, 129.4, 130.76, 131.36, 133.1, 133.5, 140.3, 151.2, 153.6, 190.1. MS: *m/z* = 430.54 [M]^+^; analysis for C_22_H_21_Cl_2_N_3_O_2_: C (61.40) H (4.92) N (9.76)%; found C (61.60) H (4.93) N (9.75)%.

*4-(3,4-Dimethoxyphenyl)-5-[4-(piperidin-1-yl)benzoyl]-3,4-dihydropyrimidin-2(1H)-one***(7)**: colour: brown; yield: 70%; m.p.: 145–147 °C; UV *λ*max (methanol) = 434 nm; IR (KBr) cm^−1^: 3478 (N–H), 2788 (ArC-H), 1634 (C = O), 1596 (C = O), 1567 (C = C); ^1^H NMR (500 MHz, DMSO-d_6_): *δ* = 1.56 (8H, s, 4 × –CH_2_, *piperidine*), 3.28 (2H, s, –CH, *piperidine*), 3.7 (6H, s, 2 × –OCH_3_), 5.42 (1H, s, H-4), 6.83–6.84 (4H, m, Ar-H), 7.0 (1H, s, NH, D_2_O exchange), 6.89–7.46 (8H, m, Ar-H), 7.73 (1H, s, =CH), 9.18 (1H, s, –CONH, D_2_O exchange); ^13^C NMR (125.76 MHz, DMSO-d_6_): *δ* = 15.6, 19.0, 24.4, 25.3, 48.0, 49.0, 53.7, 55.9, 56.5, 63.3, 110.9, 112.1, 112.9, 113.9, 118.7, 127.3, 130.7, 148.5, 149.9, 152.0, 153.6, 190.6; MS: *m/z* = 422.18 [M + 1]^+^; analysis for C_24_H_27_N_3_O_4_: C (68.39) H (6.46) N (9.97)%; found C (68.57) H (6.47) N (9.99)%.

*4-(2-Methoxyphenyl)-5-[4-(piperidin-1-yl) benzoyl]-3,4-dihydropyrimidin-2(1H)-one***(8)**: colour: yellow; yield: 50%; m.p.: 160–162 °C; UV *λ*max (methanol) = 429 nm; IR (KBr) cm^−1^: 3441 (N–H), 2931 (ArC-H), 1634 (C = O), 1595 (C = O), 1530 (C = C); ^1^H NMR (500 MHz, DMSO-d_6_): *δ* = 1.57 (8H, s, 4 × –CH_2_, *piperidine*), 2.73 (1H, s, –CH, *piperidine*), 2.87 (1H, s, –CH, *piperidine*), 3.81 (3H, s, –OCH_3_), 5.73 (1H, s, H-4), 6.87–7.25 (8H, m, Ar-H), 7.31 (1H, s, NH, D_2_O exchange), 7.45 (1H, s, =CH), 9.13 (1H, s, –CONH, D_2_O exchange); ^13^C NMR (125.76 MHz, DMSO-d_6_): *δ* = 24.4, 25.3, 48.5, 49.6, 55.9, 112.9, 130.7, 152.2, 153.3, 190.1; MS: *m/z* = 391.00 [M]^+^; analysis C_23_H_25_N_3_O_3_: C (70.57) H (6.44) N (10.73)%; found C (70.82) H (6.43) N (10.75)%.

*4-(4-Hydroxyphenyl)-5-[4-(piperidin-1-yl) benzoyl]-3,4-dihydropyrimidin-2(1H)-one***(9)**: colour: brown; yield: 45%; m.p.: 210–212 °C; UV *λ*max (methanol) = 404 nm; IR (KBr) cm^−1^: 3270 (N–H), 2930 (ArC-H), 1670 (C = O), 1593 (C = O), 1508 (C = C); ^1^H NMR (500 MHz, DMSO-d_6_): *δ* = 1.55 (8H, s, 4 × –CH_2_, *piperidine*), 2.73 (1H, s, –CH, *piperidine*), 2.88 (1H, s, –CH, *piperidine*), 5.37 (1H, s, H-4), 6.71–6.99 (8H, m, Ar-H), 7.14 (1H, s, NH, D_2_O exchange), 7.95 (1H, s, =CH), 9.20 (1H, s, –CONH, D_2_O exchange), 9.90 (1H, s, OH, D_2_O exchange); ^13^C NMR (125.76 MHz, DMSO-d_6_): *δ* = 15.6, 24.4, 25.3, 31.2, 36.2, 48.0, 48.5, 53.6, 65.4, 113.4, 113.8, 115.5, 116.3, 127.3, 128.0, 130.7, 132.5, 135.3, 138.7, 152.1, 153.9, 157.1, 162.7, 190.6; MS: *m/z* = 379.61 [M + 2]^+^; analysis for C_22_H_23_N_3_O_3_: C (70.01) H (6.14) N (11.13)%; found C (70.25) H (6.15) N (11.11)%.

*4-(3-Hydroxyphenyl)-5-[4-(piperidin-1-yl) benzoyl]-3,4-dihydropyrimidin-2(1H)-one***(10)**: colour: black; yield: 45%; m.p.: 190–192 °C; UV *λ*max (methanol) = 420 nm; IR (KBr) cm^−1^: 3200 (N–H), 2930 (ArC-H), 1654 (C = O), 1636 (C = O), 1600 (C = C); ^1^H NMR (500 MHz, DMSO-d_6_): *δ* = 1.55 (8H, s, 4 × –CH_2_, *piperidine*), 2.73 (1H, s, –CH, *piperidine*), 2.87 (1H, s, –CH, *piperidine*), 5.40 (1H, s, H-4), 6.7–6.9 (8H, m, Ar-H), 7.0 (1H, s, NH, D_2_O exchange), 7.95 (1H, s, =CH), 9.30 (1H, s, –CONH, D_2_O exchange), 9.70 (1H, s, OH, D_2_O exchange); ^13^C NMR (125.76 MHz, DMSO-d_6_): *δ* = 15.6, 21.6, 24.4, 25.3, 25.7, 31.1, 36.2, 48.0, 48.5, 53.9, 65.4, 113.2, 113.6, 113.8, 114.7, 117.3, 127.2, 129.8, 130.7, 138.9, 146.1, 152.2, 153.6, 157.9, 162.7, 172.7, 190.5; MS: *m/z* = 376.94 [M]^+^; analysis for C_22_H_23_N_3_O_3_: C (70.01) H (6.14) N (11.13)%; found C (70.24) H (6.14) N (11.10)%.

*4-(4-Dimethylamino phenyl)-5-[4-(piperidin-1-yl) benzoyl]-3,4-dihydropyrimidin-2(1H)-one***(11)**: colour: black; yield: 40%; m.p.: 185–187 °C; UV *λ*max (methanol) = 435; IR (KBr) cm^−1^: 3479 (N–H), 2788 (ArC-H), 1634 (C = O), 1596 (C = O), 1567 (C = C); ^1^H NMR (500 MHz, DMSO-d_6_): *δ* = 1.56 (8H, s, 4 × –CH_2_, *piperidine*), 2.81 (2H, s, –CH, *piperidine*), 3.0 (6H, s, -N(CH_3_)_2_, 5.30 (1H, s, H-4), 6.7–6.9 (8H, m, Ar-H), 7.0 (1H, s, NH, D_2_O exchange), 7.69 (1H, s, =CH), 9.67 (1H, s, –CONH, D_2_O exchange); ^13^C NMR (125.76 MHz, DMSO-d_6_): *δ* = 15.6, 24.4, 25.3, 48.0, 48.5, 65.3, 111.5, 113.2, 130.9, 131.9, 132.8, 154.6, 190.2; MS: *m/z* = 405.20 [M + 1]^+^; analysis for C_24_H_28_N_4_O_2_: C (71.26) H (6.98) N (13.85)%; found C (71.01) H (6.96) N (13.84)%.

*4-(3-Methoxyphenyl)-5-[4-(piperidin-1-yl) benzoyl]-3,4-dihydropyrimidin-2(1H)-one***(12)**: colour: yellow; yield: 50%; m.p.: 160–162 °C; UV *λ*max (methanol) = 432; IR (KBr) cm^−1^: 3246 (N–H), 2929 (ArC-H), 1701 (C = O), 1654 (C = O), 1600 (C = C); ^1^H NMR (500 MHz, DMSO-d_6_): *δ* = 1.57 (8H, s, 4 × –CH_2_, *piperidine*), 2.7 (1H, s, –CH, *piperidine*), 2.80 (1H, s, –CH, *piperidine*), 3.72 (3H, s, –OCH_3_), 5.43 (1H, s, H-4), 6.82–6.93 (6H, m, Ar-H), 7.0 (1H, s, NH, D_2_O exchange), 7.25–7.44 (2H, m, Ar-H), 7.78 (1H, s, =CH), 9.18 (1H, s, –CONH, D_2_O exchange); MS: *m/z* = 392.40 [M + 1]^+^; analysis for C_23_H_25_N_3_O_3_: C (70.57) H (6.44) N (10.73)%; found C (70.77) H (6.43) N (10.71)%.

*4-(4-Ethoxyphenyl)-5-[4-(piperidin-1-yl) benzoyl]-3,4-dihydropyrimidin-2(1H)-one***(13)**: colour: yellow; yield: 55%; m.p.: 200–202 °C; UV *λ*max (methanol) = 444 nm; IR (KBr) cm^−1^: 3270 (N–H), 2800 (ArC-H), 1672 (C = O), 1631 (C = O), 1600 (C = C); ^1^H NMR (500 MHz, DMSO-d_6_): *δ* = 1.30 (3H, t, *J* = 7.0 Hz, CH_3_), 1.57 (8H, s, 4 × –CH_2_, *piperidine*), 2.74 (1H, s, –CH, *piperidine*), 2.89 (1H, s, –CH, piperidine), 3.98 (2H, q, *J* = 9.0 Hz, -OCH_2_), 5.38 (1H, s, H-4), 6.86-6.93 (4H, m, Ar-H), 6.97 (1H, s, NH, D_2_O exchange), 7.20 (4H, m, Ar-H), 7.69 (1H, s, =CH), 9.11 (1H, s, –CONH, D_2_O exchange); ^13^C NMR (125.76 MHz, DMSO-d_6_): *δ* = 15.1, 24.0, 25.3, 48.0, 50.0, 65.0, 111.5, 113.2, 130.9, 131.9, 132.8, 154.6, 158.0, 162.0, 190.3; MS: *m/z* = 405.00 [M]^+^; analysis for C_24_H_27_N_3_O_3_: C (71.09) H (6.71) N (10.36)%; found C (71.34) H (6.72) N (10.34)%.

*4-(2,4,5-Trimethoxyphenyl)-5-[4-(piperidin-1-yl) benzoyl]-3,4-dihydropyrimidin-2(1H)-one***(14)**: colour: brown; yield: 60%; m.p.:155–157 °C; UV *λ*max (methanol) = 449 nm; IR (KBr) cm^−1^: 3300 (N–H), 2800 (ArC-H), 1701 (C = O), 1686 (C = O), 1654 (C = C); ^1^H NMR (500 MHz, DMSO-d_6_): *δ* = 1.57 (8H, s, 4 × –CH_2_, *piperidine*), 2.70 (1H, s, –CH, *piperidine*), 2.80 (1H, s, –CH, *piperidine*), 3.71 (9H, s, 3 × –OCH_3_), 5.62 (1H, s, H-4), 6.74–6.96 (6H, m, Ar-H), 7.0 (1H, s, NH, D_2_O exchange), 7.50 (1H, s, =CH), 9.20 (1H, s, –CONH, D_2_O exchange); ^13^C NMR (125.76 MHz, DMSO-d_6_): *δ* = 15.0, 19.1, 24.5, 25.3, 48.0, 48.5, 50.0, 56.2, 60.6, 61.3, 65.3, 108.1, 114.4, 113.2, 113.5, 123.5, 127.6, 129.8, 130.5, 132.6, 142.0, 151.5, 153.3, 153.5, 190.1; MS: *m/z* = 451.00 [M]^+^; analysis for C_25_H_29_N_3_O_5_: C (66.50) H (6.47) N (9.31)%; found C (66.70) H (6.48) N (9.33)%.

*4-(2,3,4-Trimethoxyphenyl)-5-[4-(piperidin-1-yl) benzoyl]-3,4-dihydropyrimidin-2(1H)-one***(15)**: colour: brown; yield: 57%; m.p.:125–127 °C; UV *λ*max (methanol) = 441 nm; IR (KBr) cm^−1^: 3478 (N–H), 2852 (ArC-H), 1634 (C = O), 1596 (C = O), 1567 (C = C); ^1^H NMR (500 MHz, DMSO-d_6_): *δ* = 1.56 (8H, s, 4 × –CH_2_, *piperidine*), 2.70 (1H, s, –CH, *piperidine*), 2.80 (1H, s, –CH, *piperidine*), 3.70 (9H, s, 3 × –OCH_3_), 5.64 (1H, s, H-4), 6.75–6.97 (6H, m, Ar-H), 7.0 (1H, s, NH, D_2_O exchange), 7.44 (1H, s, =CH), 9.20 (1H, s, –CONH, D_2_O exchange); ^13^C NMR (125.76 MHz, DMSO-d_6_): *δ* = 15.6, 19.0, 24.4, 25.3, 48.0, 48.5, 49.8, 56.2, 60.6, 61.3, 65.3, 108.1, 112.4, 113.2, 113.8, 123.0, 127.4, 129.9, 130.6, 132.3, 142.0, 151.5, 153.3, 153.5, 190.4; MS: *m/z* = 452.08 [M + 1]^+^; analysis for C_25_H_29_N_3_O_5_: C (66.50) H (6.47) N (9.31)%; found C (66.30) H (6.46) N (9.29)%.

*4-(3,4,5-Trimethoxyphenyl)-5-[4-(piperidin-1-yl) benzoyl]-3,4-dihydropyrimidin-2(1H)-one***(16)**: colour: brown; yield: 60%; m.p.: 135–137 °C; UV *λ*max (methanol) = 441 nm; IR (KBr) cm^−1^: 3236 (N–H), 2933 (ArC-H), 1701 (C = O), 1650 (C = O), 1610 (C = C); ^1^H NMR (500 MHz, DMSO-d_6_): *δ* = 1.55 (8H, s, 4 × –CH_2_, *piperidine*), 2.70 (1H, s, –CH, *piperidine*), 2.80 (1H, s, –CH, *piperidine*), 3.6 (9H, s, 3 × –OCH_3_), 5.40 (1H, s, H-4), 6.65–6.93 (6H, m, Ar-H), 7.0 (1H, s, NH, D_2_O exchange), 7.5 (1H, s, =CH), 9.2 (1H, s, –CONH, D_2_O exchange); ^13^C NMR (125.76 MHz, DMSO-d_6_): *δ* = 19.5, 24.4, 25.2, 25.3, 47.9, 48.5, 54.1, 56.2, 60.3, 65.3, 104.1, 112.4, 113.8, 127.2, 130.7, 137.2, 140.1, 152.9, 153.3, 153.6, 190.6; MS: *m/z* = 452.40 [M + 1]^+^; analysis for C_25_H_29_N_3_O_5_: C (66.50) H (6.47) N (9.31)%; found C (66.70) H (6.48) N (9.32)%.

*4-(2,4,6-Trimethoxyphenyl)-5-[4-(piperidin-1-yl) benzoyl]-3,4-dihydropyrimidin-2(1H)-one***(17)**: colour: brown; yield: 60%; m.p.: 140–142 °C; UV *λ*max (methanol) = 428 nm; IR (KBr) cm^−1^: 3300 (N–H), 2930 (ArC-H), 1685 (C = O), 1654 (C = O), 1595 (C = C); ^1^H NMR (500 MHz, DMSO-d_6_): *δ* = 1.54 (8H, s, 4 × –CH_2_, *piperidine*), 2.7 (1H, s, –CH, *piperidine*), 2.80 (1H, s, –CH, *piperidine*), 3.70 (9H, s, 3 × –OCH_3_), 5.79 (1H, s, H-4), 6.90–6.93 (6H, m, Ar-H), 7.0 (1H, s, NH, D_2_O exchange), 7.51 (1H, s, =CH), 9.21 (1H, s, –CONH, D_2_O exchange); ^13^C NMR (125.76 MHz, DMSO-d_6_): *δ* = 15.6, 19.0, 24.4, 25.3, 48.0, 48.5, 49.5, 56.0, 56.5, 60.6, 65.4, 112.4, 112.5, 113.8, 120.2, 124.3, 127.3, 130.7, 130.9, 137.6, 139.5, 146.5, 151.8, 152.9, 153.5, 190.3; MS: *m/z* = 453.92 [M + 2]^+^; analysis for C_25_H_29_N_3_O_5_: C (66.50) H (6.47) N (9.31)%; found C (66.35) H (6.46) N (9.30)%.

*4-(2,4-Dimethoxyphenyl)-5-[4-(piperidin-1-yl) benzoyl]-3,4-dihydropyrimidin-2(1H)-one***(18)**: colour: brown; yield: 55%; m.p.: 135–137 °C; UV *λ*max (methanol) = 419 nm; IR (KBr) cm^−1^: 3270 (N–H), 2900 (ArC-H), 1670 (C = O), 1635 (C = O), 1621 (C = C); ^1^H NMR (500 MHz, DMSO-d_6_): *δ* = 1.57 (8H, s, 4 × –CH_2_, *piperidine*), 2.70 (1H, s, –CH, *piperidine*), 2.80 (1H, s, –CH, *piperidine*), 3.83 (6H, s, 2 × –OCH_3_), 5.64 (1H, s, H-4), 6.44–6.93 (7H, m, Ar-H), 7.0 (1H, s, NH, D_2_O exchange), 8.0 (1H, s, =CH), 9.07 (1H, s, –CONH, D_2_O exchange); ^13^C NMR (125.76 MHz, DMSO-d_6_): *δ* = 15.6, 24.4, 25.3, 48.0, 48.5, 49.3, 55.6, 55.9, 65.4, 99.1, 104.8, 111.7, 113.2, 113.2, 124.0, 127.4, 128.6, 130.7, 132.8, 139.6, 152.2, 153.5, 158.3, 160.4, 190.4; MS: *m/z* = 421.67 [M]^+^; analysis for: C (68.39) H (6.46) N (9.97)%; found C (66.45) H (6.47) N (9.95)%.

## In vivo anti-ulcer activity

### Evaluation of anti-ulcer activity and gastric secretion in rats

Albino Wistar rats, weighing (150–200 g), were obtained from the animal house of College of Pharmacy, King Saud University (Riyadh, Saudi Arabia). All the animals were kept in laboratory conditions for 1 week, so that they will get acclimatised. The animals were randomly divided into groups of six rats each. Compounds (**1–18**) were given orally or intraperitoneally. The stomachs were removed after the rats were sacrificed and opened along the greater curvature. The animal protocol used in this study was approved by the Research Ethics Committee of College of Pharmacy, King Saud University.

### Gastric lesions induced by ethanol

Albino Wistar rats, weighing (150–200 g), were divided into different groups. Animals were administered test drugs or standard drug. After 1 h, 1 ml of 80% ethanol was administered orally to each animal^23^.

### Gastric lesions induced by necrotising agents (cytoprotection)

Necrotising agent, 1 ml each (80% ethanol, 0.2 mol/l NaOH or 25% NaCl), was administered to animals. Compounds (**3**, **8**, **11** and **15**) were given half an hour prior to the administration of necrotising agents. The animals were sacrificed and examined for stomach ulcers after 1 h of the administration of necrotising agents.

### Gastric lesions induced by indomethacin

Suspension of indomethacin in 1.0% of carboxymethylcellulose (CMC) in water (6 mg/ml) at a dose of (30 mg/kg) body weight was administered orally. Control rats were treated with vehicle. Compounds (**3**, **8**, **11** and **15**) were given half an hour prior to indomethacin administration at a dose of 12.5, 25 and 50 mg/kg^24^.

### Hypothermic restraint stress-induced ulcers

Thirty minutes after the oral administration of compounds (**3**, **8**, **11**, and **15**), 12.5, 25 and 50 mg/kg of the rats were restrained in cages and kept inside a refrigerator for 3 h^25^.

### Pylorus-ligated rats

Pylorus ligation under ether anaesthesia was carried out. Intraperitoneal administration of compounds (**3**, **8**, **11** and **15**) was performed immediately after pylorus ligation. After 6 h, animals were sacrificed^26^.

### Determination of gastric wall mucus (GWM)

GWM was performed according to the modified procedure^27^.

### Estimation of non-protein sulfhydryls (NP-SH) MDA and total protein (TP)

Gastric mucosal non-protein sulfhydryls, MDA and TP were measured according to the reported method^28^.

### Determination of LD_50_

The Karber method was used for the LD_50_ determination of most active compounds^29^.

### Histopathological evaluation

Histopathological examination of gastric tissue was performed to study the anti-ulcer activity of compounds (**3**, **8**, **11** and **15**).

## Results and discussions

### Chemistry

**Scheme 1.**
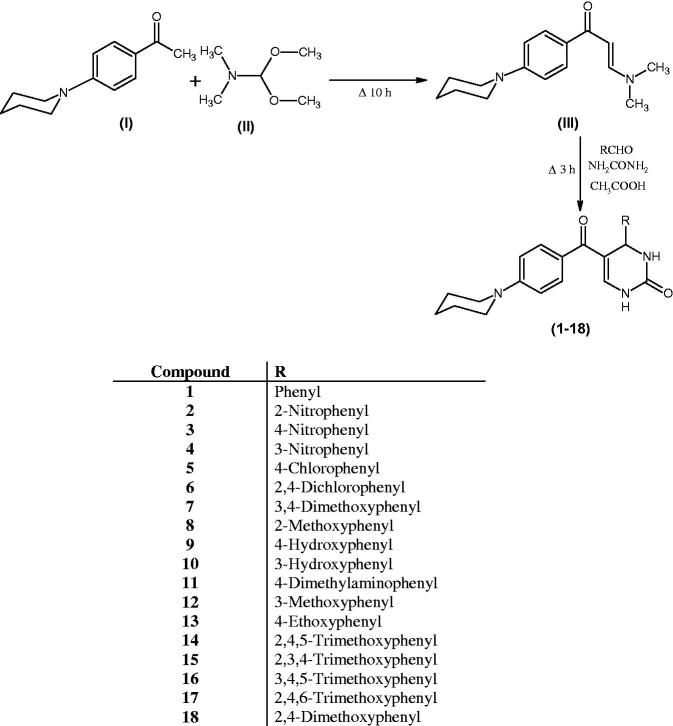
Synthetic route of compounds **(1–18)**.

As shown in Scheme 1, enaminone **(III)**, 3-(dimethylamino)-1-[4-(piperidin-1-yl) phenyl] prop-2-en-1-one was synthesised by refluxing 1-[4-(piperidin-1-yl) phenyl] ethan-1-one **(I)** with DMF-DMA **(II)** under solvent free condition for 10 h.

**Figure 1.**
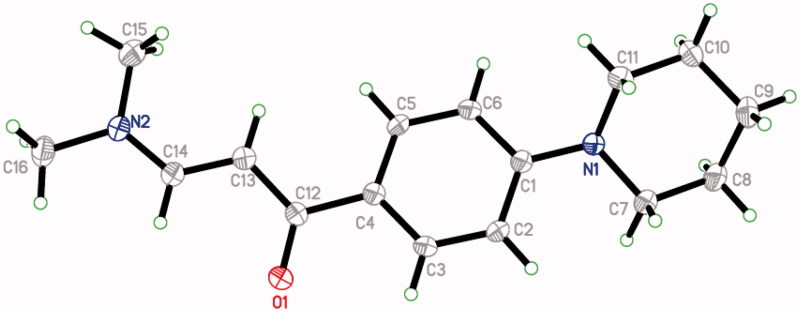
Single crystal X-ray structure of enaminone **(III)**.

Six protons of piperidine were obtained as a singlet at *δ* 1.58 ppm and four piperidine protons appeared at *δ* 3.0 ppm. Two singlet peaks, at *δ* 2.89 and 3.17 ppm, were obtained due to the *N*,*N*-dimethyl protons and two doublet peaks at *δ* 5.79 and 7.65 ppm (*J* = 12.5 Hz) were obtained due to the ethylenic protons in ^1^H NMR^30^. Aromatic protons were found around *δ* 6.91–7.78 ppm. The enaminone **(III)** existed in the *E*-configuration. A single crystal X-ray structure also confirmed the 3D structure of enaminone **(III)** (Figure 1).

**Figure 2.**
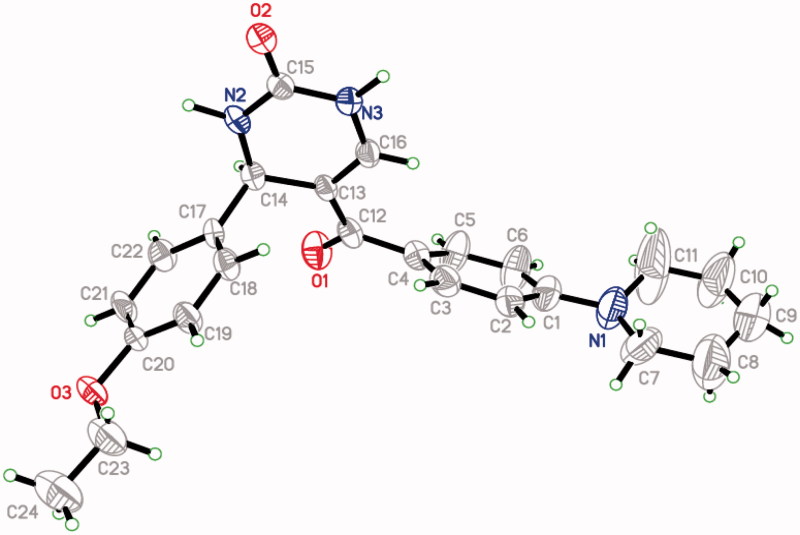
Single crystal X-ray structure of compound **13**.

A reaction mixture of substituted benzaldehyde (0.01 mol), enaminone, 3-(dimethylamino)-1-[4-(piperidin-1-yl) phenyl] prop-2-en-1-one **(III)** (0.01 mol), urea (0.01 mol) and glacial acetic acid (10 ml) was refluxed for 3 h. The products were obtained by pouring the reaction mixture in cold water. The precipitates (**1–18)** thus formed were collected by vacuum filtration. The products were washed several times with cold water. Re-crystallisation of products was performed in glacial acetic acid. All of the compounds presented the D_2_O exchangeable broad singlet at *δ* 6.97–7.31 ppm and *δ* 9.07–9.67 ppm corresponding to the two NH protons. Eight protons (4 × CH_2_) of piperidine moiety were observed at *δ* 1.54–1.57 ppm. Two other piperidine protons were observed at *δ* 2.70–3.44 and *δ* 2.80–3.48 ppm^31^. The H-4 and = CH protons of dihydropyrimidinone moiety were observed at *δ* 5.37–6.11 and 7.45–8.21 ppm, respectively. The presence of all carbon atoms for compounds was confirmed by ^13^C NMR spectra. The CH_2_ carbons of piperidine were obtained at around *δ* 24, 25, 48 and 53 ppm. The carbonyl group (C = O) peak was observed at around 190. Molecular weight of compounds was confirmed by mass spectra. All the compounds gave molecular ion peak respective to their molecular weights. The detailed spectral results of ^1^H NMR, ^13^C NMR spectra and mass spectra are given in the experimental part. The spectral and analytical data confirmed the composition of the synthesised compounds (**1–18**). The single crystal X-ray structure confirms the 3D structure of dihydropyrimidinone derivative **13** (Figure 2).

### Biological activity *in vivo*

In our first phase study, we screened all the synthesised compounds (**1–18**) at graded doses (12.5, 25 and 50 mg/kg, p.o.) in 80% ethanol induced gastric ulcer model with ranitidine (50 mg/kg, p.o.) as reference drug. The screening results are summarised in Table 1. Among the synthesised compounds, **3**, **8**, **11** and **15** exhibited significant protection. It gives us the impetus to further explore their anti-ulcer effects in different anti-ulcer models.

**Table 1.** The effect of compounds on gastric lesions induced by 80% ethanol (mean ± SE).

				Compounds					
	80% EtOH	Ranitidine 50 (mg/kg)		12.5 (mg/kg)		25 (mg/kg)		50 (mg/kg)	
Compounds	Mean ± SE	Mean ± SE	% Change	Mean ± SE	% Change	Mean ± SE	% Change	Mean ± SE	% Change
**1**	7.5 ± 0.28	1.75 ± 0.47***	76.66	7.25 ± 0.47	–	6.75 ± 0.25	–	6.25 ± 0.47*	–
**2**	7.00 ± 0.40	2.00 ± 0.40***	71.42	6.00 ± 0.16	14.2	5.50 ± 0.28*	21.4	5.00 ± 0.16**	28.5
**3**	7.50 ± 0.28	2.00 ± 0.40	73.3	5.0 ± 0.40***	33.33	3.5 ± 0.2***	53.3	3.00 ± 0.4***	60.0
**4**	6.75 ± 0.25	2.25 ± 0.47***	66.6	6.75 ± 0.25	–	5.50 ± 0.08	–	6.25 ± 0.25	7.40
**5**	7.0 ± 0.40	2.0 ± 0.4***	71.4	7.0 ± 0.4	–	6.5 ± 0.28	7.1	6.25 ± 0.47	10.7
**6**	7.0 ± 0.40	2.5 ± 0.28***	64.2	7.25 ± 0.25	–	6.5 ± 0.2	7.1	6.25 ± 0.25	10.7
**7**	7.5 ± 0.28	2.75 ± 0.25***	63.3	6.75 ± 0.25	10	5.5 ± 0.2***	26.6	5.0 ± 0.4***	33.3
**8**	7.5 ± 0.28	2.2 ± 0.4***	70	5.0 ± 0.4***	33.3	3.7 ± 0.4***	50	3.5 ± 0.2***	53.3
**9**	7.0 ± 0.4	2.2 ± 0.6***	67.8	7.2 ± 0.25	–	6.5 ±	2	6.0 ± 0.4	–
**10**	7.0 ± 0.4	2.0 ± 0.5***	71.4	6.0 ± 0.4	14	5.75 ± 0.2*	17.8	5.5 ± 0.2*	21.4
**11**	7.7 ± 0.25	1.7 ± 0.4***	77.4	6.0 ± 0.4**	22.5	4.0 ± 0.1***	48.3	3.0 ± 0.4***	61.2
**12**	7.0 ± 0.4	2.7 ± 0.2***	60.7	7.0 ± 0.4	–	6.5 ± 0.2	7.1	6.25 ± 0.4	10.7
**13**	7.0 ± 0.40	1.7 ± 0.4***	75	7.0 ± 0.4	–	6.5 ± 0.2	7.1	6.0 ± 0.4	14.2
**14**	7.2 ± 0.25	1.70.4***	75.8	6.5 ± 0.2	10.3	5.25 ± 0.4**	27.5	4.5 ± 0.2***	37.9
**15**	7.7 ± 0.2	2.5 ± 0.2***	67.7	5.0 ± 0.4***	35.4	3.5 ± 0.2***	54.8	2.5 ± 0.2***	67.7
**16**	7.0 ± 0.4	2.0 ± 0.4***	71.4	6.5 ± 0.2	7.1	5.75 ± 0.4	17.8	5.5 ± 0.2*	21.4
**17**	7.2 ± 0.2	2.75 ± 0.2***	62	7.00 ± 0.4	–	5.2 ± 0.4**	27.5	4.5 ± 0.4**	37.9
**18**	7.7 ± 0.2	2.0 ± 0.4***	74.1	7.2 ± 0.2	6.4	6.5 ± 0.2*	16.1	6.0 ± 0.4**	22.5

Six rats were used in each group.

* *p* < .05,

** *p* < .01,

*** *p* < .001 vs. control group, Student’s *t*-test.

The animals were treated with 80% ethanol, 0.2 mol/l NaOH and 25% NaCl, which resulted in gastric lesions in the stomach in all the control animals. The ulcer index in 80% ethanol, 0.2 mol/l NaOH and 25% NaCl was 7.66 ± 0.21, 7.33 ± 0.21 and 6.83 ± 0.30, respectively, in the control animals after the 1-h administration of necrotising agents. Pre-treatment of animals with compounds **3**, **8**, **11** and **15** at doses of 12.5, 25, 50 mg/kg produced significant results. Compound **15** (50 mg/kg) was found to be most active as anti-ulcer agent with ulcer index in 80% ethanol, 0.2 mol/l NaOH and 25% NaCl as 2.16 ± 0.30, 1.33 ± 0.42 and 1.66 ± 0.33, *p* < 0.001, respectively (Table 2).

**Table 2.** The effect of compounds on gastric lesions induced by necrotising agents (mean ± SE).

		Ulcer index		
Treatment	Dose (mg/kg, i.p.)	80% EtOH	0.2 mol/l NaOH	25% NaCl
**Control**	1 ml	7.66 ± 0.21	7.33 ± 0.21	6.83 ± 0.30
**Ranitidine (standard)**	50	1.50 ± 0.22***	1.00 ± 0.36***	1.16 ± 0.30***
**3**	12.5	6.83 ± 0.30*	4.50 ± 0.22***	5.16 ± 0.47*
**3**	25	4.16 ± 0.30***	2.66 ± 0.33***	2.83 ± 0.30***
**3**	50	3.00 ± 0.36***	1.83 ± 0.40***	1.66 ± 0.33***
**8**	12.5	7.00 ± 0.36	6.66 ± 0.33	6.00 ± 0.25
**8**	25	6.50 ± 0.42*	5.33 ± 0.71*	5.00 ± 0.44**
**8**	50	5.83 ± 0.30***	3.83 ± 0.30***	3.33 ± 0.30***
**11**	12.5	7.16 ± 0.30	6.33 ± 0.42	6.00 ± 0.36
**11**	25	6.16 ± 0.30**	3.66 ± 0.21***	4.83 ± 0.40**
**11**	50	4.83 ± 0.30***	3.66 ± 0.33***	3.83 ± 0.30***
**15**	12.5	4.66 ± 0.33***	3.50 ± 0.22***	3.66 ± 0.33***
**15**	25	2.66 ± 0.33***	2.16 ± 0.30***	2.66 ± 0.33***
**15**	50	2.16 ± 0.30***	1.33 ± 0.42***	1.66 ± 0.33***

Six rats were used in each group.

* *p* < .05,

** *p* < .01,

*** *p* < .001 vs. control group, Student’s *t*-test.

NSAIDs are considered to be responsible for peptic ulcer in humans due to suppression of PGE_2_ biosynthesis and depletion of mucus. The administration of indomethacin (30 mg/kg) orally induced gastric damage of animals. The compounds **3**, **8**, **11** and **15** presented significant results especially compounds **3** and **15** with ulcer index of 12.66 and 14.50 respectively, which provides a proof, regarding the cytoprotective nature of these compounds. Compound **3** was found to be most active anti-ulcer agent in this test (Table 3).

**Table 3.** The effect of compounds on indomethacin-induced gastric mucosal lesions (mean ± SE).

Treatment	Dose (mg/kg, i.p.)	Ulcer index
**Control (indomethacin)**	30	35.66 ± 1.05
**Ranitidine (standard)**	50	8.50 ± 0.56***
**3**	12.5	29.83 ± 1.66*
**3**	25	20.50 ± 1.52***
**3**	50	12.66 ± 1.28***
**8**	12.5	33.00 ± 1.52
**8**	25	29.50 ± 1.58*
**8**	50	28.66 ± 1.45**
**11**	12.5	33.00 ± 1.03
**11**	25	30.66 ± 1.28*
**11**	50	28.33 ± 1.78**
**15**	12.5	26.33 ± 1.30***
**15**	25	21.50 ± 1.33***
**15**	50	14.50 ± 1.64***

Six rats were used in each group.

* *p* < .05,

** *p* < .01,

*** *p* < .001 vs. control (indomethacin only) group, Student’s *t*-test.

Ulcer formation by hypothermic restraint stress was inhibited significantly by compounds **3** and **15** at the dose of 50 mg/kg. However, compound **15** was found to be most effective at dose of 50 mg/kg with intraluminal bleeding and gastric lesion ulcer index of 1.33 ± 0.33 and 12.33 ± 0.84, respectively. Compound **3** was observed to show similar activity as compound **15** at the same dose of 50 mg/kg (Table 4).

**Table 4.** The effect of compounds on hypothermic restraint stress-induced intraluminal bleeding and gastric lesion in rats (mean ± SE).

Treatments (*n* = 6)	Dose (mg/kg, i.p.)	Intraluminal bleeding score	Gastric lesion ulcer index
**Control**		4.16 ± 0.30	33.00 ± 1.26
**Ranitidine (standard)**	50	0.83 ± 0.30***	9.66 ± 0.95***
**3**	12.5	2.83 ± 0.30*	24.16 ± 1.70**
**3**	25	1.50 ± 0.22***	17.66 ± 0.76***
**3**	50	1.16 ± 0.30***	13.83 ± 0.60***
**8**	12.5	3.50 ± 0.42	29.83 ± 1.50
**8**	25	3.33 ± 0.21*	27.66 ± 1.60*
**8**	50	2.50 ± 0.42*	18.66 ± 0.55***
**11**	12.5	3.66 ± 0.33	29.83 ± 1.51
**11**	25	2.66 ± 0.33**	29.00 ± 1.21*
**11**	50	2.00 ± 0.36***	21.00 ± 0.51***
**15**	12.5	2.16 ± 0.30***	25.66 ± 1.08**
**15**	25	1.66 ± 0.21***	16.66 ± 0.33***
**15**	50	1.33 ± 0.33***	12.33 ± 0.84***

Six rats were used in each group.

* *p* < .05,

** *p* < .01,

*** *p* < .001 control (distilled water) group, Student’s *t*-test.

In the experiment of pylorus ligation, a large amount of gastric acid secretion were obtained (11.23 ± 0.18 ml), titratable acidity was found to be 173.88 ± 5.12 mEq/l and ulcer index was recorded as 3.33 ± 0.21 in the control group. Compounds **3** and **15** significantly reduced the gastric secretion, titratable acidity and ulcer index at the dose dependent manner. Compound **15** at the dose of 50 mg/kg was found to be most effective in reducing gastric secretion, titratable acidity and ulcer index formation 4.76 ± 0.23 ml, 73.33 ± 2.43 mEq/l and 1.00 ± 0.36 respectively as compared to the standard drug ranitidine (Table 5).

**Table 5.** The effect of compounds on gastric secretion, acidity and gastric lesion index in pylorus-ligated shay rats (mean ± SE).

Treatment	Dose (mg/kg, i.p.)	Volume of gastric content (ml)	Titratable acidity (mEq/l)	Ulcer index
**Control**	–	11.23 ± 0.18	173.88 ± 5.12	3.33 ± 0.21
**Ranitidine (standard)**	50	4.06 ± 0.18***	58.88 ± 1.85***	0.50 ± 0.22***
**3**	12.5	9.03 ± 0.24***	153.88 ± 5.40*	2.33 ± 0.33*
**3**	25	6.31 ± 0.25***	97.77 ± 2.93***	1.83 ± 0.30**
**3**	50	4.63 ± 0.22***	84.84 ± 2.38***	1.16 ± 0.30***
**8**	12.5	10.50 ± 0.34	161.11 ± 4.36	3.16 ± 0.30
**8**	25	9.36 ± 1.22**	133.33 ± 3.22***	2.50 ± 0.22*
**8**	50	6.46 ± 0.16***	115.00 ± 5.75***	2.00 ± 0.13*
**11**	12.5	10.20 ± 0.29*	160.55 ± 3.48	2.83 ± 0.30
**11**	25	7.35 ± 0.19***	141.11 ± 6.30**	2.50 ± 0.42
**11**	50	6.63 ± 0.21***	116.66 ± 4.63***	2.33 ± 0.21**
**15**	12.5	6.68 ± 0.18***	116.11 ± 2.64***	1.83 ± 0.30**
**15**	25	5.50 ± 0.24***	86.11 ± 3.98***	1.50 ± 0.22***
**15**	50	4.76 ± 0.23***	73.33 ± 2.43***	1.00 ± 0.36***

Six rats were used in each group.

* *p* < .05,

** *p* < .01,

*** *p* < .001 vs. control (distilled water) group, Student’s *t*-test.

**Figure 3.**
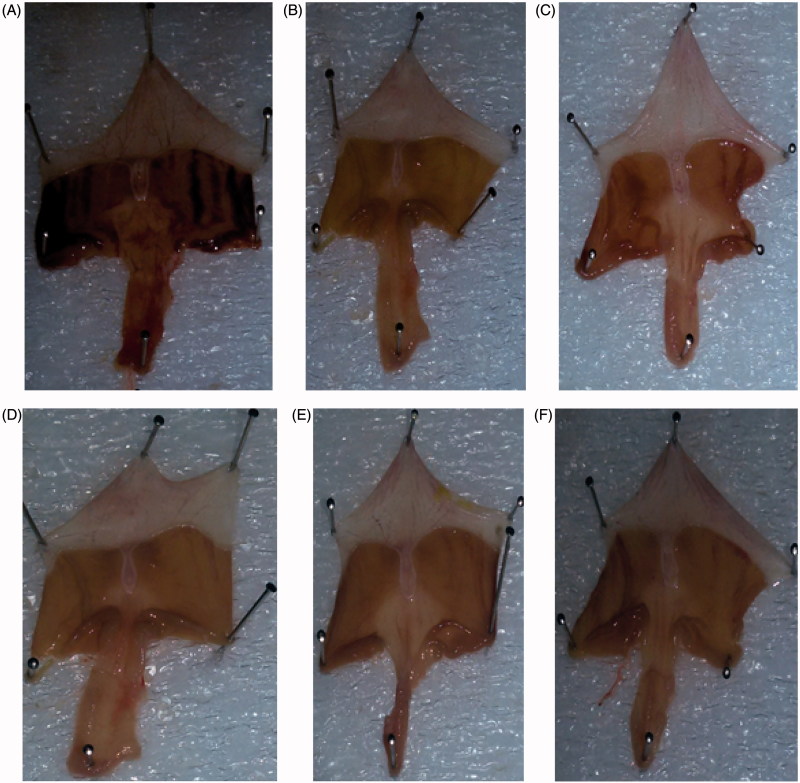
(A) Treatment with 80% ethanol only, showing mucosal ulceration. (B) Treatment with ranitidine (50 mg/kg) showing normal mucosa. (C) Treatment with compound **3** (50 mg/kg) showing intact mucosa with mild ulceration. (D) Treatment with compound **8** (50 mg/kg) showing intact normal mucosa. (E) Treatment with compound **11** (50 mg/kg) showing intact normal mucosa. (F) Treatment with compound **15** (50 mg/kg) showing intact normal mucosa.

The administration of ethanol induced a significant damage to the mucosa. Treatment with 80% ethanol resulted in gastric mucosal ulceration (Figure 3(A)), ranitidine pre-treatment showed the normal gastric mucosa (Figure 3(B)), compound **3** (50 mg/kg) pre-treatment presented intact mucosa with mild ulceration (Figure 3(C)), pre-treatment with compounds **8**, **11** and **15** (50 mg/kg) each showed intact normal gastric mucosa (Figure 3(D–F)).

There is a significant reduction in the Alcian blue binding of gastric mucus (201 ± 8.32 µg/g) of tissue in animals treated with 80% ethanol as compared to control group (276.53 ± 10.19 µg/g). Pre-treatment of animals with compounds **3**, **8**, **11** and **15** at different doses produced dose dependent effects. Compounds **3** and **15** were found to be most effective. Compound **15** at the dose of (50 mg/kg) significantly enhances the Alician blue binding capacity of gastric mucosa (275.32 ± 5.37 µg/g), *p* < 0.001 (Table 6).

**Table 6.** The effect of compounds on the change in gastric wall mucus in stomach tissue induced by 80% ethanol (mean ± SE).

Treatment	Dose (mg/kg, i.p.)	Gastric wall mucus (mean ± SE, µg/g)
**Control (normal)**	–	276.53 ± 10.19
**80% EtOH**	1 ml	201.91 ± 8.32***^,^^a^
**Ranitidine (standard)**	50	287.24 ± 10.70***^,^^b^
**3**	12.5	242.08 ± 4.03*^,^^b^
**3**	25	241.66 ± 6.91**^,^^b^
**3**	50	256.18 ± 8.39***^,^^b^
**8**	12.5	206.39 ± 7.18^b^
**8**	25	212.00 ± 6.40^b^
**8**	50	244.65 ± 5.36**^,^^b^
**11**	12.5	192.87 ± 12.84^b^
**11**	25	224.88 ± 4.64*^,^^b^
**11**	50	237.36 ± 3.31**^,^^b^
**15**	12.5	231.78 ± 4.77*^,^^b^
**15**	25	248.09 ± 7.69**^,^^b^
**15**	50	275.32 ± 5.37***^,^^b^

Six rats were used in each groups.

* *p* < .05,

** *p* < .01,

*** *p* < .001 vs. control (80% ethanol only) group, Student’s *t*-test.

a As compared to the control group.

b As compared to 80% ethanol only group.

**Figure 4.**
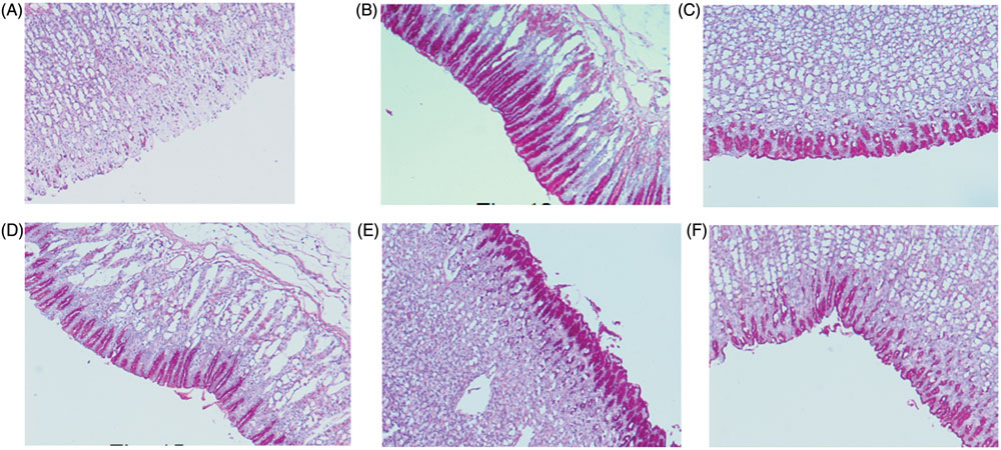
Light micrographs showing the effect of compounds **3**, **8**, **11** and **15** on ethanol-induced gastric lesions of rats. (A) Treatment with ethanol (PAS); (B) pre-treatment with standard drug ranitidine (50 mg/kg) (PAS); (C) pre-treatment with compound **3** (50 mg/kg) (PAS); (D) pre-treatment with compound **8** (50 mg/kg) (PAS); (E) pre-treatment with compound **11** (50 mg/kg) (PAS); (F) pre-treatment with compound **15** (50 mg/kg) (PAS).

The glycogen level of the control and the pre-treated animal were also checked using the Periodic acid-Schiff (PAS). The ulcers induced by ethanol causes extensive gastric mucosal injury. Moreover, they exhibit haemorrhagic and necrotic lesions, which infiltrate into the mucosa and cause oedema and leukocyte infiltration. However, the pre-treatment with compounds **3**, **8**, **11** and **15** resulting in expansion of mucus gel layer that with continuous PAS-positive that lines the gastric mucosal surface (Figure 4). The magenta staining colour is exhibited with the compounds **3**, **8**, **11** and **15** pre-treated groups. The tissue has a normal glandular pattern and mild leucocyte infiltration. On the other hand, the gastric specimen from the control did not exhibit the magenta staining colour. As shown in Figure 4(A), the ethanol-induced ulcer exhibits pervasive injury to the gastric mucosa. The pre-treatment with ranitidine protects the gastric mucosa (Figure 4(B)). The compounds **3**, **8**, **11** and **15** pre-treated rats exhibited a significant decrease in ulcer index and less mucosal damage (Figure 4(C–F)). These results clearly indicate that compounds **3**, **8**, **11** and **15** have gastro-protective activity. Mucus production by gastric mucosa increased gradually in the experimental rats pre-treated with compounds **3**, **8**, **11** and **15**. Gastric mucus plays a crucial role in gastro-protection. The pre-treatment with compounds **3**, **8**, **11** and **15** significantly augmented the gastro-protective activity, with enhancement of the free mucus when compared to the mucus of ulcer control animals. Thus, compounds **3**, **8**, **11** and **15** have gastro-protective activity against ethanol induced gastric ulcer by improving mucosal content.

MDA levels in the gastric mucosa were significantly increased in ethanol only treated then in control group (7.42 ± 0.30 nmol/g; 1.14 ± 0.06 nmol/g). Compounds **15** (50 mg/kg) significantly reduced the MDA content (1.90 ± 0.06 nmol/g). Similar results were obtained for compound **3**. The NP-SH level in control group was found to be 5.03 ± 0.10 nmol/g of tissue, which was significantly reduced to 3.22 ± 0.20 nmol/g of tissue following the 80% ethanol administration. Pre-treatment of animals with compounds **3**, **8**, **11** and **15** significantly replenished the ethanol induced depletion of NP-SH. Compounds **3** and **15** at the dose of 50 mg/kg produced highly significant results 4.56 ± 0.17 and 4.92 ± 0.30, respectively, higher than the standard drug ranitidine (4.24 ± 0.15). The level of TP in the gastric mucosa of control group was 122.55 ± 3.23 g/l, which was significantly decreased to 47.50 ± 2.08 g/l following 80% ethanol administration. Pre-treatment of animals with tested compounds significantly improved the levels of TP. Compounds **15** and **3** at the dose of 50 mg/kg produced significant results (96.60 ± 1.18 g/ml and 95.80 ± 1.51 g/ml), respectively, in comparison to the standard drug ranitidine (104.59 ± 1.59 g/ml) (Table 7).

**Table 7.** The effect of compounds on the levels of MDA, NP-SH and TP in stomach tissue induced by 80% ethanol (mean ± SE).

Treatment	Dose (mg/kg, i.p.)	MDA (nmol/g)	NP-SH (nmol/g)	Total protein (g/l)
**Control (normal)**	–	1.14 ± 0.06	5.03 ± 0.10	122.55 ± 3.23
**80% EtOH**	1 ml	7.42 ± 0.30***^,^^a^	3.22 ± 0.20***^,^^a^	47.50 ± 2.08***^,^^a^
**Ranitidine (standard)**	50	1.65 ± 0.02***^,^^b^	4.24 ± 0.15**^,^^b^	104.59 ± 1.59***^,^^b^
**3**	12.5	4.47 ± 0.44***^,^^b^	3.15 ± 0.20^b^	58.68 ± 3.19*^,^^b^
**3**	25	3.07 ± 0.16***^,^^b^	4.23 ± 0.23**^,^^b^	74.65 ± 3.79***^,^^b^
**3**	50	1.95 ± 0.05***^,^^b^	4.56 ± 0.17***^,^^b^	95.80 ± 1.51***^,^^b^
**8**	12.5	6.63 ± 0.26^b^	3.49 ± 0.16^b^	45.90 ± 1.14^b^
**8**	25	4.83 ± 0.24***^,^^b^	3.61 ± 0.12^b^	55.88 ± 1.71*^,^^b^
**8**	50	3.75 ± 0.07***^b^	4.61 ± 0.27**^,^^b^	66.26 ± 1.47***
**11**	12.5	5.16 ± 0.22***^,^^b^	2.93 ± 0.11^b^	53.89 ± 1.48*^,^^b^
**11**	25	3.99 ± 0.17***^,^^b^	3.40 ± 0.18^b^	64.27 ± 2.08***^,^^b^
**11**	50	3.36 ± 0.08***^,^^b^	4.35 ± 0.11***^,^^b^	72.25 ± 1.43***^,^^b^
**15**	12.5	3.51 ± 0.08***^,^^b^	3.38 ± 0.07^b^	71.45 ± 1.43***^,^^b^
**15**	25	2.72 ± 0.10***^,^^b^	4.29 ± 0.24**^,^^b^	81.43 ± 3.65***^,^^b^
**15**	50	1.90 ± 0.06***^,^^b^	4.92 ± 0.30***^,^^b^	96.60 ± 1.18***^,^^b^

Six rats were used in each groups,

* *p* < .05,

** *p* < .01,

*** *p* < .001 vs. control (80% ethanol only) group, Student’s *t*-test.

a As compared to the control group.

b As compared to 80% ethanol only group.

### Toxicity of compounds

Karber method was used to determine the LD_50_ of compounds **3**, **8** and **15**. A 24-h observation was made for the toxicity symptoms and mortality. The dead animals were counted at the end of the study and the LD_100_ was calculated. The LD_50_ of compounds **3**, **8** and **15** were found to be 125, 55.5 and 116.5 mg/kg, respectively (Table 8).

**Table 8.** Determination of LD_50_ of active compounds by Karber method.

	Group	Dose (mg/kg)	Number of animals	DD (a)	Dead	MM (b)	Pro.(a*b)
**Compound 3**	1	5	10		0		
	2	25	10	20	0	0	0
	3	50	10	25	2	1	25
	4	100	10	50	5	3.5	175
	5	200	10	100	8	6.5	650
	6	300	10	100	10	9	900
						Total product	1750
						**LD_50_ = 125 mg/kg**	
**Compound 8**	1	5	10		0		
	2	25	10	20	2	1	20
	3	50	10	25	6	4	100
	4	100	10	50	9	7.5	375
	5	200	10	100	10	9.5	950
	6	300	10	100	10	10	1000
	–	–	–	–	–	Total product	2445
						**LD_50_ = 55.5 mg/kg**	
**Compound 15**	1	5	10		0	0	
	2	25	10	20	1	0.5	10
	3	50	10	25	3	2	50
	4	100	10	50	4	3.5	175
	5	200	10	100	9	6.5	650
	6	300	10	100	10	9.5	950
	–	–	–	–	–	Total product	1835
						**LD_50_ = 116.5 mg/kg**	

DD: dose difference; MM: mean mortality; Factor = last lethal dose – (total product/number of animals).

### Structure activity relationship (SAR)

The design of new compounds was based on hybrid approach. A series of compounds containing dihydropyrimidinone and piperidine were synthesised and screened for anti-ulcer activity. Structural modifications were done not only to obtain derivatives with higher activity, but also to collect data regarding SAR. We showed that the presences of pharmacophores (dihydropyrimidinone and piperidine) are both essential for the activity. Compounds **3** (**R** = 4-nitrophenyl substitution), **8** (**R** = 2-methoxyphenyl), **11** (**R** = *N*-dimethylaminophenyl) and **15** (**R** = 2,3,4-trimethoxyphenyl) substitutions were found to be most active compounds of the series.

## Conclusion

A series of novel dihydropyrimidinone and piperidine scaffold hybrids were synthesised, characterised by spectral data and screened for their anti-ulcer activity in several *in vivo* ulcer models. The newly synthesised hybrids displayed significant gastro protective effect by inhibiting the formation of ulcers induced by 80% ethanol. Four compounds **3**, **8**, **11** and **15** were found to most potent compounds of the series. These compounds were further evaluated for anti-ulcer activity by different *in vivo* anti-ulcer models in animals. The anti-ulcer action of the active compounds appears to be due to both anti-secretary and gastro protective effect. The gastro protective action was mainly due to secretion of mucus. Compound **15** was found to be highly potent compounds of the series. Additional studies on lead compound **15** will result in a new orally active candidate.
